# Association between Gestational Weight Gain and Maternal and Birth Outcomes in Northern Ghana

**DOI:** 10.1155/2024/5526942

**Published:** 2024-05-02

**Authors:** John Lapah Niyi, Zhihui Li, Fidelis Zumah

**Affiliations:** ^1^Ghana Health Service, Gushegu Municipal Health Directorate, Gushegu, Ghana; ^2^Vanke School of Public Health, Tsinghua University, 100084 Beijing, China; ^3^Institute for Health China, Tsinghua University, 100084 Beijing, China; ^4^School of Collective Intelligence, Mohammed VI Polytechnic University (UM6P), Rabat, Morocco; ^5^University of Ghana Medical Centre Ltd, Legon, Accra, Ghana

## Abstract

**Background:**

Although inappropriate gestational weight gain is considered closely related to adverse maternal and birth outcomes globally, little evidence was found in low- and middle-income countries. *Study Objectives*. This study is aimed at identifying the determinants of gestational weight gain and examine the association between gestational weight gain and maternal and birth outcomes in the Northern Region of Ghana. *Study Methods*. The study used a facility-based cross-sectional study design involving 611 antenatal and delivery records in Tatale district, Tamale west, and Gushegu municipal hospitals. A two-stage sampling method involving cluster and simple random sampling was employed. Descriptive statistical analysis and measures of central tendency were used to describe the sample. The multinomial logistic regression model was used to determine the determinants of gestational weight gain and its association with maternal and birth outcomes.

**Results:**

Among the 611 women included in the study, 516 (84.45%) had inadequate gestational weight gain, and 19 (3.11%) had excessive gestational weight gain. The gestational weight gain ranged from 2 kg to 25 kg with a mean of 7.26 ± 3.70 kg. The risk factor for inadequate gestational weight gain was low prepregnancy BMI (adjusted odds ratio (AOR) = 1.33, 95% CI = 1.18 − 2.57, *P* = 0.002). Pregnant women who had inadequate gestational weight gain were significantly less likely to deliver through caesarean section (AOR = 0.27, 95% CI = 0.12 − 0.61, *P* = 0.002), and those who had excessive weight gain were more likely to undergo caesarean section (AOR = 19.81, 95% CI = 5.38 − 72.91, *P* = 0.001). The odds of premature delivery (birth < 37 weeks) among pregnant women with inadequate weight gain were 2.88 (95% CI = 1.27 − 6.50, P = 0.011). Furthermore, subjects who had excessive weight gain were 43.80 times more likely to give birth to babies with macrosomia (95% CI = 7.07 − 271.23, *P* = 0.001).

**Conclusion:**

Inappropriate gestational weight gain is prevalent in Ghana, which is associated with caesarean section, preterm delivery, delivery complications, and macrosomia. Urgent policy interventions are needed to improve on the frequent monitoring and management of gestational weight gain of pregnant women till term.

## 1. Background

Optimal weight gain during pregnancy is critical for the health of women and their babies during and after pregnancy and is positively related to ideal birth outcomes [1]. Although there are no established known guidelines for the classification of gestational weight gain by the World Health Organization (WHO), the Institute of Medicine (IoM) in the United States of America established guidelines for gestational weight gain in 2009. The guideline stipulates that pregnant women with prepregnancy body mass index (BMI) classified as underweight, normal weight, overweight, and obese are expected to gain an overall weight of 12.7-18.1 kg, 11.5-16.0 kg, 7.0-11.5 kg, and 5.0-9.0 kg, respectively [2]. There is a rich body of literature from developed countries showing that an optimal gestational weight gain is linked with lower maternal mortality, induction of labor, foetal macrosomia, premature birth, and perinatal deaths.

There is evidence from high-income countries including China, Europe, the United States of America, and Brazil showing the associated risk factors of inappropriate gestational weight gain and its association with maternal and birth outcomes [3–11]. However, evidence in low- and middle-income countries (LMICs) specifically in the context of Ghana is limited. Hence, this study was conducted to address this gap pertaining to the Ghanaian context.

Research findings point to a high prevalence of inappropriate gestational weight gain (inadequate and excessive gestational weight gain) worldwide. In a population-based study conducted in the United States, the prevalence of low gestational weight gain and excessive gestational weight gain was found to be 21.6% and 45.6%, respectively [3]. Additionally, [12] reported in a systematic review and meta-analysis of over a million pregnant women that 47% and 23% of pregnant women had excessive and inadequate gestational weight gain, respectively. Some studies in LMICs, including Ghana, have reported a high prevalence of inappropriate gestational weight gain. A multicountry study conducted in LMICs found that all WHO Africa regions involved in the study had a mean gestational weight gain below the minimum gestational weight gain, with Sub-Saharan Africa and North Africa along with the Middle East reporting the lowest gestational weight gains at 6.64 kg and 6.80 kg, respectively [13]. In Malawi, the prevalence of inadequate gestational weight gain was found to be 80.2% [14]. Excessive gestational weight gain was also increasingly reported in LMICs. For example, there were 55.5% of the women in South Africa observed to have excessive gestational weight gain [15].

Previous studies have explored the various determinants of gestational weight gain. The following factors were found to be significantly associated with inappropriate gestational weight gain: prepregnancy BMI classified as obese (AOR = 3.0, 95% CI = 1.6 − 6.3, *P* < 0.001), high calorie intake (AOR = 4.49, 95% CI = 1.61 − 12.46, *P* < 0.001), age within 30-40 years (AOR = 2.88, 95% CI = 1.13 − 7.35, *P* = 0.027), and smoking/former smoker (AOR = 5.18, 95% CI = 1.68 − 16.52, *P* = 0.005) [16–18].

Furthermore, inadequate gestational weight gain has been reported as a risk factor for multiple adverse birth outcomes, including low birth weight (relative risk (RR) = 4.15, 95% confidence interval (CI) = 1.10 − 15.40, P = 0.03), preterm birth (adjusted odds ratio (AOR) = 3.53, 95% CI = 1.96 − 6.37, *P* < 0.001), and perinatal death [3, 9, 19–21]. Additionally, excessive gestational weight gain was associated with negative maternal and neonatal outcomes, such as the high risk of emergency caesarian section, induction of labor, episiotomy, macrosomia, and neonatal hypoglyceamia [4, 11, 20, 22]. In previous studies, excessive gestational weight gain was reported to be associated with macrosomia (AOR = 2.20, 95% CI = 1.44 − 2.93, *P* < 0.001), caesarean section (AOR = 1.45, 95% CI = 1.13 − 1.87, *P* = 0.004), and neonatal hypoglycaemia (AOR = 3.30, 95% CI = 1.20 − 12.00, *P* = 0.023) [9]. However, a majority of the evidence stemmed from developed countries such as the United States of America, China, Brazil, and Europe [7, 11, 23]. Comparing these findings to LMICs may be challenging due to differences in socioeconomic and cultural contexts. To the best of our knowledge, there is limited research available in the context of LMICs, including Ghana [24]. Previous research conducted in Ghana provided limited insights into the connection between inadequate and excessive weight gain and their impact on both maternal and birth outcomes [22, 25]. To date, the only study conducted within the context of northern Ghana focusing on the relationship between gestational weight gain and maternal outcomes exclusively utilized data from three urban hospitals [22]. Consequently, the findings of this study may not be inferred to encompass rural and periurban areas owing to the variations in socioeconomic and cultural background, hence, the need for this current study. This study will contribute to the scientific evidence base for clinical and public health practice in the context of the Northern Region and Ghana as a whole.

This study is aimed at identifying the determinants of gestational weight gain and the association between gestational weight gain and maternal and birth outcomes using secondary data collected from three randomly selected health facilities in the Northern Region of Ghana. The findings and recommendations of this study will serve as a guide in the prevention and management of risk factors for inappropriate gestational weight gain and its management during pregnancy to curb maternal and birth outcomes associated with inappropriate gestational weight gain.

## 2. Methodology

### 2.1. Study Design

We adopted a facility-based cross-sectional study design to examine the determinants of gestational weight gain and the association between gestational weight gain and maternal and birth outcomes in selected health facilities in the Northern Region of Ghana. The study area is in the Northern Region of Ghana. The Northern Region is one of the sixteen regions in Ghana. It is located in the north of the country, covering an area of 70,384 square kilometers or 31 percent of Ghana's land area until December 2018 when the Savannah Region and North East Region were created from it. The Northern Region is divided into 10 districts, 5 municipalities, and 1 metropolitan with Tamale being the regional capital. The Northern Region has a low population density and is bordered on the north by the North East Region, on the east by the eastern Ghana-Togo international border, on the south by the Oti region, and on the west by the Savannah region. The region currently has a total population of 2,310,939 with an annual population growth rate of 3.7% based on the 2021 Ghana population and housing census. The total population of women in fertile age (WIFA) is 554,625. The predominant economic activity in the region is agriculture. About 75% of the economically active population is engaged in agricultural activities [26]. The Northern Region currently has 19 hospitals. The region recorded a total of 90,663 antenatal care (ANC) registrants in 2021. The total number of deliveries recorded in 2021 was 63,041 [27].

### 2.2. Sampling Method and Data Collection

The study was conducted in three randomly selected district/municipal hospitals in the Northern Region. The selected facilities were Gushegu Municipal Hospital, Tatale/Sanguli District Hospital, and Tamale West Hospital. A multistage sampling method was employed. At the first stage, districts/municipals in the Northern Region were classified into three clusters, that is, urban, periurban, and rural districts/municipals. Names of all district/municipal hospitals in the three clusters were written on pieces of paper, folded, and kept in 3 separate bowls based on the clusters. In the last stage, a district/municipal hospital was randomly selected from each bowl after the bowls containing names of the district/municipal hospitals in each cluster have been shaken. This sampling method was used to ensure that records of women with different socioeconomic backgrounds and social groups in the region are included in the study. Since gestational weight gain can be affected by socioeconomic status. The data were extracted using a data extraction sheet designed to capture the demographic characteristics, gestational weight at the first and third trimesters, and maternal and birth outcomes of the women at the selected district/municipal hospitals. The data extraction sheet was used to collect variables of interest from ANC records and delivery registers.

### 2.3. Inclusion and Exclusion Criteria

This study included records of all deliveries in the delivery register in the three [3] selected hospitals in the Northern Region from January to December 2021 and had their ANC records available in the same hospitals. Subjects with multiple (2 or more) births were also excluded since the record of only one newborn was needed to match each maternal record because multiple births could affect outcomes. Records that do not have valid data on 1st trimester weight and other key variables were excluded. Deliveries before 28 weeks of gestation were also excluded.

**Figure 1:**
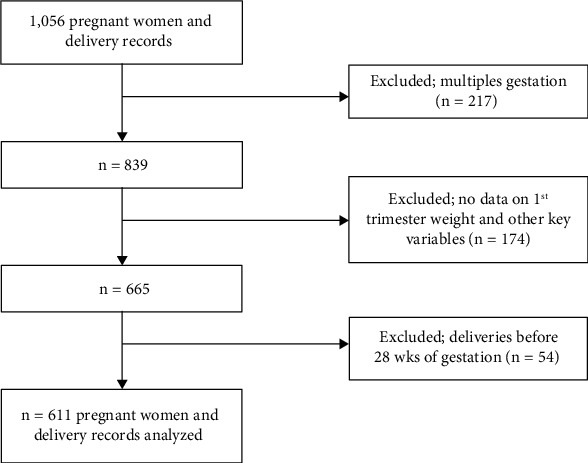
Sample selection flow chart.

As shown in Figure 1, there were a total of 1,056 ANC and delivery records of the participants. 455 records were excluded as they met the exclusion criteria. In the end, 611 records were analyzed.

As presented in Table 1, participants who had gestational weight gain less than 12.5 kg, 11.5 kg, 7.0 kg, and 5.0 kg with prepregnancy BMI of underweight, normal weight, overweight, and obese correspondingly were classified to have inadequate weight gain. Also, participants who had gestational weight gain greater than 18.0 kg, 16.0 kg, 11.5 kg, and 9.0 kg with prepregnancy BMI of underweight, normal weight, overweight, and obese, respectively, were classified to have excessive weight gain.

### 2.4. Statistical Analysis

Descriptive statistical analysis was performed to describe the study sample in relation to the relevant variables. The results were presented using tables and figures in frequencies and percentages where necessary appropriate measures of central tendency (mean, median, and mode) and dispersion (standard deviation and ranges) were calculated on variables with respect to the study objectives.

Both crude and adjusted multinomial logistic regression model was employed to quantify and test the robustness of the association between gestational weight gain and maternal and birth outcomes.

EpiData Entry Client version 4.6.0.2 was used for data entry and validation. Consistency and plausibility checks were done after the data entry to ensure that errors are reduced. The data was then exported to STATA version 14.0 for analysis.

### 2.5. Ethical Considerations

Ethical clearance for this study was waived as the secondary data used for this study is readily available if granted permission to access it. Permission was granted by the Health Directorates of the selected districts and the selected hospitals to use data from their facilities after the study was explained to them.

No individual identifying information such as names and addresses was linked to data analysis and dissemination of the study findings. This was necessary to ensure the privacy and confidentiality of client records.

## 3. Results

### 3.1. Demographic Characteristics of Study Subjects

Table 2 shows the demographic characteristics of the pregnant women. Regarding the age distribution, the results revealed that the mean age of the respondents was 27.04 years (standard deviation (SD): 6.90), with ages ranging from 14 to 50 years. Most participants (50.90%) were in the age group of 20-29 years, followed by 31.59% in the 30-39-year age group. Pregnant women aged 40 years and older represented the smallest group, accounting for 4.75% of the total. In terms of education, 62.03% had no formal education, while only 2.78% had tertiary-level education. Regarding occupation, 57.12% were self-employed or in apprenticeship training, 39.61% were unemployed, and only 3.27% were fully employed as public/civil servants. Maritally, the majority (90.83%) were married.

In terms of gravidity, the mean gravidity was 2.90 (±1.88), ranging from 1 to 10. The majority (74.44%) had been pregnant at least once before, while 25.66% were first-time pregnant. Regarding parity, the mean parity was 2.23 (±1.78), with a range of 1 to 9. Most pregnant women (54.64%) had given birth more than once, 33.06% were experiencing their first pregnancy, and a smaller portion (12.27%) had never given birth.

The mean prepregnancy BMI for the 611 participants was 22.13 (±3.78) kg/m^2^, with the highest and lowest recorded BMIs being 38.20 kg/m^2^ and 11.90 kg/m^2^, respectively. The majority (68.74%) had a normal BMI, while 3.76% were obese, and 13.58% were underweight, with 13.91% being overweight. Additionally, the mean gestational weight gain among the pregnant women was 7.26 (±3.70) kg, ranging from 2 kg to 26 kg. The majority (84.45%) had inadequate weight gain, while 3.11% had excessive weight gain.

As presented in Table 3, among pregnant women with normal gestational weight gain, 23.68% had an underweight prepregnancy BMI, while 14.47% were obese. In contrast, among those with inadequate gestational weight gain, 12.40% were underweight, and only 1.55% were obese. For participants with excessive gestational weight gain, 21.05% were obese, and 5.26% were underweight. Prepregnancy BMI was found to be a statistically significant factor.

### 3.2. Factors Associated with Gestational Weight Gain

Table 4 shows the results of a multivariate multinomial logistic regression model analysis which presented the associations between risk factors and gestational weight gain. We found that prepregnancy BMI was a significant determinant of gestational weight gain. As compared to normal prepregnancy BMI, underweight pregnant women were 33% more likely to have inadequate gestational weight gain (AOR = 1.33, 95% CI = 1.18 − 2.57, *P* = 0.002) whereas pregnant women who were obese were 72% less likely to gain inadequate gestational weight (AOR = 0.28, 95% CI = 0.15 − 0.53, *P* = 0.001) in reference to normal weight gain.

The remaining factors including maternal age, education, gravidity, and parity were not significant determinants of gestational weight gain.

### 3.3. Association between Gestational Weight Gain and Pregnancy and Birth Outcomes

The multinomial logistic regression analysis presented in Table 5 shows the association between pregnancy outcome variables and gestational weight gain. The results indicated that pregnant women who delivered by caesarean section were 73% less likely to have inadequate weight gain at 95% CI = 0.12 − 0.61, *P* = 0.002, and 19.81 times more likely to have excessive weight gain (95% CI = 5.38 − 72.91, *P* = 0.001) as compared to those who had spontaneous vaginal delivery.

Furthermore, compared to participants without delivery complications, those who had delivery complications are 8.47 times more likely to have excessive weight gain compared to normal weight gain (95% CI = 1.29 − 55.33, *P* = 0.026).

The findings also showed that the odds of preterm delivery among pregnant women who had inadequate weight gain was 2.88 (95% CI = 1.27 − 6.50, *P* = 0.011) as compared to normal-term delivery. Furthermore, pregnant women who delivered postterm as compared to those who delivered at normal term were 91% less likely to have inadequate weight gain with reference to normal weight gain (*AOR* = 0.09, 95% *CI* = 0.03 − 0.32, *P* = 0.001).

Pregnant women who had excessive weight gain were 43.80 (95% CI = 7.07 − 271.23, *P* = 0.001) times more likely to give birth to macrosomia babies as compared to normal birth weight babies.

## 4. Discussion

### 4.1. Determinants of Gestational Weight Gain

We conducted a comprehensive and geographically representative study and analysis of routine data in 3 hospitals selected from rural, periurban, and urban districts in northern Ghana. This study sought to determine the factors associated with gestational weight gain and the association between gestational weight gain and pregnancy outcome. The findings showed that inappropriate gestational weight gain had varying impact on maternal and neonatal outcomes. The prominent findings in this study revealed that prepregnancy BMI was a significant risk factor for gestational weight gain. Furthermore, gestational weight gain was significantly associated with mode of delivery, delivery complications, term of delivery, and foetal macrosomia.

In the present study, underweight prepregnancy BMI increased the risk of inadequate gestational weight gain by 33% while overweight/obese prepregnancy BMI decreased the risk by 77%. This finding was strongly supported by previous studies [28, 29]. For instance, among low-income Hispanic women in the United States of America, decreased odds (0.36) of inadequate gestational weight gain was observed among women who were overweight or obese before pregnancy [30]. In the same vain, another study observed that underweight women had an increased risk of inadequate gestational weight gain [31]. Notably, maternal nutritional status before and during pregnancy is a major contributing factor to prepregnancy BMI and gestational weight gain [28]; hence, nutritional deficiencies coupled with the high range of optimum gestational weight gain for underweight women in the IoM guidelines may account for their increased risk of insufficient gestational weight gain. However, Mbu et al. found that underweight prepregnancy BMI was positively associated with excessive gestational weight gain [28]. These contrary findings may result from the different categorization method used by Mbu et al. which had only two groups for gestational weight gain, that is, normal and excessive. Consequently, these modifiable risk factors are detrimental to the attainment of optimal gestational weight. Hence, it is imperative to adapt initiatives aimed at enhancing normal BMI before pregnancy such as appropriate dietary practices and physical activities [15, 32]. This present study, however, was unable to demonstrate that age of mother, marital status, and educational status are significant determinants of gestational weight gain, consistent with earlier studies [1, 22, 28].

### 4.2. Association between Gestational Weight Gain and Maternal and Birth Outcome

This study highlights the association between gestational weight gain and maternal and birth outcome. It has been reported that excessive gestation weight gain predisposes women to delivery complications [28, 33, 34], which concurs with the findings in our study. Such associated complications include prolonged labor, high risk of arrest disorders, narrow birth passage due to accumulation of soft tissues, intra- and postpartum haemorrhage, and eclampsia [28]. These preventable complications are among the leading causes of maternal and neonatal deaths in LMICs including Ghana [35]. Consequently, those women have to undergo emergency caesarean section in order to limit or prevent the negative impact of these complications [28, 33]. This has further been affirmed in this study and supported by previous research [5, 11, 28]. Interestingly, caesarean section was significantly high in women with greater weight gain during pregnancy. In Cameroon for instance, pregnant women who had excessive weight gain were over 3 times at greater risk to undergo emergency caesarean section [28]. On this basis, it can be deduced that the higher risk of delivery complications and foetal macrosomia among women with excessive gestational weight may account for the increased odds of caesarean section.

Gestational weight gain below the IoM recommendations has been observed to be a significant risk factor of preterm delivery. In China, for example, insufficient weight gain was significantly associated with a high probability of preterm delivery [9, 36]. In accordance with these previous studies, we observed that inadequate weight gain during pregnancy increased the risk of preterm births but was found to be a protective factor for postterm delivery. Although these results are consistent with previous studies as indicated, they differ from some other published research [37, 38]. For instance, Huang et al. [38] reported that increased gestational weight gain above IoM recommendations increases the risk of preterm birth. However, these findings relate to gestational weight gain only in the 3^rd^ trimester of gestation, whereas in the current study, gestational weight gain was obtained from 1^st^ to 3^rd^ trimester of gestation. It seems possible that inadequate gestational weight gain may be reflective of nutritional deficiencies particularly during early gestation, which could affect plasma volume expansion and maternal tissue development to support the foetus to term, resulting to preterm delivery [39, 40]. Indeed, preterm delivery poses major health complications to babies and increases the risk of neonatal and infant mortality. Therefore, measures must be put in place to reduce the higher prevalence of certain risk factors of preterm delivery including inadequate gestational weight gain as reported in this study. We further observed that compared to normal weight gain, weight gain above the IoM guidelines was associated with a higher risk of foetal macrosomia. Several other studies conducted in China, Cameroon, Nigeria, and Sweden reported comparable results where foetal macrosomia was positively associated with excessive gestational weight gain [5, 9, 21, 28]. It has been established that foetal macrosomia is a major risk factor of childhood obesity. For example, in China, foetal macrosomia increased the risk of childhood obesity by 3.74 folds compared to normal birth weight babies [23]. Consequently, it resulted to the early onset of chronic cardiovascular diseases [23]. The negative implications of inappropriate gestational weight gain demand urgent behavioural counselling interventions for gestational weight gain to minimize the risk of these undesirable maternal and birth outcomes.

### 4.3. Limitation of the Study

This study had some limitations. First, the use of records of the subjects is available in the delivery and ANC registers. Since the data was not collected for the purposes of this current study, the data on other variables that could have enhanced the content of this study were not captured in the registers. This limitation was however minimized by extracting data on key variables that would help achieve the objectives of this study.

Secondly, some of the records had incomplete data or missing key variables, and hence, some of the records were not extracted. To cater for a limited sample size with complete records of key variables of interest, three hospitals were used to generate adequate records for this current study.

Also, gestational weight gain was classified as inadequate, normal, and excessive weight gain based on the United States of America (USA) IoM guidelines for gestational weight gain. Indeed, these guidelines were developed in the context of the US population; hence, they might not be suitable for developing countries like Ghana. Nevertheless, these guidelines have been accepted and used in LMICs including Cameroon, Nigeria, Niger, and Ghana [1, 17, 21, 28]. Therefore, the findings from the current study are comparable to similar studies conducted in similar settings.

Finally, the cross-sectional study design used made it impossible to derive causation between dependent and independent variables. In minimizing this limitation, the adjusted multinomial logistic regression model was used in this study to strengthen the association between gestational weight gain and its determinants. Also, respondent, recall, and interviewer biases did not affect this study since the researcher did not obtain data directly from the subjects but rather used medically confirmed and recorded data from registers without altering or manipulating data on exposures and outcomes.

## 5. Conclusion

This study explored the determinants of gestational weight gain and its association with maternal and birth outcomes among pregnant women in northern Ghana. Inadequate gestational weight gain was predominant among the study subjects. Prepregnancy BMI (underweight and obese) were a significant determinant of inadequate gestational weight gain. Pregnant women with inadequate gestational weight had greater odds of preterm delivery, while excessive gestational weight also increased the likelihood of caesarean section delivery and delivery complications. Finally, pregnant women with excessive gestational weight gain were more likely to deliver newborns with macrosomia. Hence, urgent policy interventions are needed for early identification and management of the risk factors. These interventions should also aimed at improving the frequent monitoring of gestational weight gain of pregnant women till term.

## Figures and Tables

**Table 1:** Classification of weight gain (adopted from the Institute of Medicine).

Prepregnancy BMI	BMI (kg/m)	Range of inadequate weight gain (kg)	Range of normal weight gain (kg)	Range of excessive weight gain (kg)
Underweight	<18.5	<12.5	12.5-18.0	>18.0
Normal weight	18.5-24.9	<11.5	11.5-16.0	>16.0
Overweight	25.0-29.9	<7.0	7.0-11.5	>11.5
Obese (includes all classes)	≥30.0	<5.0	5.0-9.0	>9.0

**Table 2:** Demographic characteristics and gestational weight gain of study subjects.

Demographic characteristics and weight gain	Frequency (*n* = 611)	Percentage
Mean age in years = 27.04 ± 6.90		
Age (years)		
Below 20	78	12.77
20-29	311	50.90
30-39	193	31.59
40 and above	29	4.75
Highest educational level		
No formal education	379	62.03
Basic	134	21.93
Secondary/technical	81	13.26
Tertiary	17	2.78
Occupation		
Public/civil servant	20	3.27
Vocational/self-employed	349	57.12
None	242	39.61
Marital status		
Married	555	90.83
Not married	56	9.17
Mean gravidity/number of all pregnancies = 2.90 ± 1.88		
Gravidity		
Primigravida	158	25.86
Multigravida	453	74.14
Mean parity/number of live births from all pregnancies = 2.33 ± 1.78		
Parity		
Nulliparous	75	12.27
Primiparous	202	33.06
Multiparous	334	54.66
Mean BMI in kg/m^2^ = 22.13 ± 3.78		
Prepregnancy BMI		
Underweight	83	13.58
Normal weight	420	68.74
Overweight	85	13.91
Obese	23	3.76
Mean gestational weight gain in kg = 7.26 ± 3.70		
Gestational weight gain		
Normal weight gain	76	12.44
Inadequate weight gain	516	84.45
Excessive weight gain	19	3.11

**Table 3:** Crosstabulation of prepregnancy BMI and gestational weight gain.

Prepregnancy BMI	Frequency	Gestational weight gain		
		Normal	Inadequate	Excessive
Underweight	83	18 (23.68)	64 (12.40)	1 (5.26)
Normal weight	420	35 (46.05)	376 (72.87)	9 (47.37)
Overweight	85	12 (15.79)	68 (13.18)	5 (26.32)
Obese	23	11 (14.47)	8 (1.55)	4 (21.05)

**Table 4:** Multinomial logistic regression showing the determinants of gestational weight gain among pregnant women using normal weight gain as the base outcome category.

Determinants	Gestational weight gain			
	Inadequate weight gain		Excessive weight gain	
	AOR (95% CI)	*P* value	AOR (95% CI)	*P* value
Age of mother (years)				
Below 20	ref		ref	
20-29	0.52 (0.13-2.07)	0.353	0.25 (0.02-2.85)	0.267
30-39	0.43 (0.09-1.87)	0.259	0.23 (0.02-3.25)	0.277
40 and above	0.92 (0.12-7.36)	0.939	1.27 (0.05-29.75)	0.883
Prepregnancy BMI				
Normal weight	ref		ref	
Underweight	1.33 (1.18-2.57)	0.002^∗^	0.22 (0.02-2.24)	0.206
Overweight/obese	0.28 (0.15-0.53)	0.001^∗^	1.87 (0.53-6.55)	0.326
Gravidity				
Primigravida	ref		ref	
Multigravida	0.43 (0.15-1.26)	0.124	0.54 (0.027-10.99)	0.690
Parity				
Nulliparous	ref		ref	
Primiparous	2.33 (0.73-7.48)	0.153	0.09 (0.01-1.36)	0.082
Multiparous	3.62 (0.99-13.28)	0.052	2.52 (0.12-54.55)	0.556
Highest educational level				
No formal education	ref		ref	
Basic	1.12 (0.56-2.26)	0.742	0.37 (0.07-1.85)	0.226
Secondary/technical/tertiary	9.44 (0.92-30.01)	0.102	13.56 (1.55-120.04)	0.061

^∗^ *P* value statistically significant at 95% CI. Confounding variables adjusted: age of mother (years), prepregnancy BMI, gravidity, parity, and highest education level.

**Table 5:** Multinomial logistic regression showing the association between maternal and birth outcome and gestational weight gain of pregnant women using normal weight gain as the base outcome category.

Maternal outcome	Gestational weight gain			
	Inadequate weight gain		Excessive weight gain	
	AOR (95% CI)	*P* value	AOR (95% CI)	*P* value
Mode of delivery				
Spontaneous vaginal delivery	ref		ref	
Caesarean section	0.27 (0.12-0.61)	0.002^∗^	19.81 (5.38-72.91)	0.001^∗^
Delivery complication				
None	ref		ref	
Had complication	0.46 (0.15-1.44)	0.185	8.47 (1.29-55.33)	0.026^∗^
Term of delivery (weeks)				
Normal term (37-41)	ref		ref	
Preterm (<37)	2.88 (1.27-6.50)	0.011^∗^	0.99 (0.18-5.64)	0.998
Postterm (<=42)	0.09 (0.03-0.32)	0.001^∗^	0.58 (0.05-6.85)	0.666
APGAR score (1 minute)				
Below normal (<7)	ref		ref	
Normal (> =7)	0.75 (0.25-2.25)	0.606	4.01 (0.29-54.85)	0.298
APGAR score (5 minutes)				
Below normal (<7)	ref		ref	
Normal (> =7)	2.52 (0.32-20.04)	0.382	0.06 (0.00-1.44)	0.082
Weight of baby (kg)				
Normal birth weight (2.5–3.9)	ref		ref	
Low birth weight (<2.5)	1.14 (0.53-2.44)	0.740	0.00	0.989
Foetal macrosomia (> =4.0)	0.17 (0.02-1.25)	0.082	43.80 (7.07-271.23)	0.001^∗^
Resuscitation				
Resuscitation provided	ref		ref	
Resuscitation not provided	2.48 (1.02-6.03)	0.066	0.46 (0.07-3.19)	0.433
Neonatal outcome				
Alive	ref		ref	
Dead	2.60 (0.22-30.40)	0.446	0.00	0.995
Stillbirth	0.92 (0.05-18.29)	0.958	0.00	0.997

^∗^ *P* value statistically significant at 95% CI. Confounding variables adjusted: mode of delivery, delivery complication, term of delivery, APGAR score, weight of baby (kg), resuscitation, and neonatal outcome.

## Data Availability

The datasets supporting the conclusion of this study are available upon reasonable request from the corresponding author.

## Abbreviations

ANC: Antenatal care
BMI: Body mass index
DHIMS2: District health information management system 2
GHS: Ghana Health Service
IoM: Institute of Medicine
I-WIP: International weight management in pregnancy
LMICs: Low- and middle-income countries
WHO: World Health Organization
WIFA: Women in fertile age.
